# Plasma cytokine levels as markers of pathogenesis and treatment response in patients with non-tuberculous mycobacterial pulmonary disease

**DOI:** 10.1590/1414-431X2024e13755

**Published:** 2024-09-06

**Authors:** Sai Zhao, Zhiqiang Zhang, Jie Xu, Zheng Zhou, Yunhua Wu, Yanhua Wu, Guosheng Jiang

**Affiliations:** 1 Department of Laboratory Medicine, Shandong Public Health Clinical Center, Shandong University, Jinan, China; 2 Immunology Department, Binzhou Medical University, Yantai, China

**Keywords:** Mycobacterium tuberculosis, Non-tuberculous mycobacteria, Cytokines, Treatment

## Abstract

We investigated the value of plasma cytokine levels as markers of pathogenesis and treatment response in patients with non-tuberculous mycobacteria (NTM) pulmonary disease. Plasma cytokine levels were measured and compared among patients with NTM pulmonary disease (n=111), tuberculosis (TB) patients (n=50), and healthy individuals (n=40). Changes during treatment were monitored at 3 and 6 months after treatment. According to the treatment response, NTM patients were classified as ‘resistance' or ‘sensitivity' responders. The results revealed that five out of twelve cytokines exhibited significantly higher levels in NTM patients compared to controls. Among these, interleukin (IL)-6 demonstrated the strongest discriminating capacity for NTM. Furthermore, when combined with IL-1β, they efficiently distinguished between NTM drug-resistant and drug-sensitive patients, as well as between NTM and TB groups. Additionally, IL-6 levels initially rose and then decreased in the NTM drug-resistant group during the six months of treatment, similar to the behavior of IL-1β in the NTM drug-sensitive group. Subgroup analyses of the sensitive group with differential treatment responses revealed an increase in IL-10 levels in the six-month treatment responders. A high IL-6/IL-10 ratio was associated with increased disease severity of NTM and TB. Collectively, combinations of various plasma cytokines, specifically IL-1β, IL-6, and IL-10, effectively distinguished NTM patients with varying mycobacterial burdens, with IL-6 and IL-10 emerging as potential biomarkers for early treatment response. The combination of IL-6 and IL-1β demonstrated the highest discriminatory value for distinguishing between NTM-resistant and NTM-sensitive groups as well as between NTM and TB groups.

## Introduction

Non-tuberculosis mycobacterium (NTM) is a collective term for mycobacteria genus other than the *Mycobacterium tuberculosis* complex and *Mycobacterium leprae* (1). The incidence of NTM infection has been increasing year by year, posing a serious threat to human health similar to tuberculosis (TB) (2). The high level of drug resistance to anti-mycobacterial drugs exhibited by NTM presents significant challenges for clinical physicians in the differential diagnosis, treatment, and prognosis of NTM disease (3). International standards commonly categorize NTM into four groups, including photochromogens, scotochromogens, non-photochromogens, and rapidly growing mycobacteria.

With the continuous development of molecular biology and immunology, emerging biotechnologies (such as gene chip technology) have played an important role in the study of NTM disease (4-6). In recent years, monitoring serum cytokines has played an important role in the occurrence and development of TB disease, but research on monitoring NTM disease is relatively rare (7).

This study systematically investigated the occurrence, development, and prognosis of NTM disease through monitoring serum cytokines. The levels of 12 cytokines [interleukin (IL)-1β, IL-2, IL-4, IL-5, IL-6, IL-8, IL-10, IL-12P70, IL-17, interferon (IFN)-γ, tumor necrosis factor (TNF)-α, and IFN-α] in the plasma of 111 patients with NTM pulmonary disease were assessed and their clinical significance in NTM pulmonary disease was evaluated. Our findings may provide an important reference for the auxiliary diagnosis, medication, and prognosis intervention of the disease.

## Material and Methods

### Study participants

We enrolled 111 patients with NTM pulmonary disease from Shandong Provincial Public Health Clinical Center from January 2019 to July 2022 as the NTM group. Additionally, 50 patients with old pulmonary TB lesions from the same period were selected as the TB group, and 40 healthy individuals were selected as the healthy control (HC) group. According to the “NTM Disease Diagnosis and Treatment Guidelines (2020)” (8), we classified NTM patients into resistant and sensitive groups. The drug-resistant group exhibited resistance to both first-line anti-tuberculosis drugs (such as isoniazid, rifampicin, ethambutol, streptomycin, and pyrazinamide) and second-line drugs (including kanamycin, amikacin, capreomycin, linezolid, prothionamide, and fluoroquinolones). In contrast, patients in the sensitive group responded effectively to both first- and second-line anti-TB medications.

The inclusion criteria for the NTM group were as follows: 1) Two separate sputum samples both showed positive NTM culture, with identification of the same pathogen, and/or NTM molecular biology tests of both samples identified the same pathogen; 2) Positive NTM culture and/or molecular biology test results in bronchial lavage fluid or bronchoalveolar lavage fluid; 3) Lung tissue examination through bronchoscopy or other methods with pathological changes characteristic of mycobacterial disease (granulomatous inflammation or positive acid-fast staining), along with positive NTM culture and/or molecular biology test in one or more sputum samples, bronchial lavage fluid, or bronchoalveolar lavage fluid; and 4) Presence of local and/or systemic symptoms and detection of NTM culture and/or molecular biology test positivity in puncture specimens or biopsy tissues from extrapulmonary tissues or organ lesion sites. The inclusion criteria for patients with old pulmonary TB lesions were: 1) Patients with direct sputum smear microscopy showing positive sputum bacteria in 2 tests; 2) Patients with one positive sputum smear and one positive culture; and 3) One positive sputum smear, confirmed by a chest X-ray with shadows of active pulmonary TB lesions.

Exclusion criteria were: 1) Patients with TB or non-TB infections combined with HIV or autoimmune diseases; and 2) TB or non-TB patients in the latent period who had no symptoms of active TB.

The baseline data of patients, such as age and gender, were recorded.

This study was approved by the Ethics Committee of Shandong Public Health Clinical Center. Informed consent was obtained from each participant.

### Flow cytometry detection of cytokines

A total of 12 cytokine detection reagent kits were purchased from Qingdao, China. Blood samples (4 mL each) were collected from all participants after overnight fasting. After centrifugation at 2350 *g* for 15 min at −4°C, the upper layer of plasma was collected. Flow cytometry was used to determine the levels of IL-1β, IL-2, IL-4, IL-5, IL-6, IL-8, IL-10, IL-12P70, IL-17, IFN-γ, TNF-α, and IFN-α in plasma. The flow cytometer FACS Caibur (BD, USA) was used.

### Statistical analysis

Statistical analysis was conducted using SPSS 13.0 (IBM, USA). Data are reported as means±SD or median (Q25, Q75). Data comparison was performed with ANOVA or Kruskal-Wallis test. ROC curve analysis was performed to assess the performance of cytokines in differentiating between NTM drug-resistant and drug-sensitive patients. The area under the ROC curve was calculated. GraphPad was used for graph plotting. Additionally, a principal component analysis was conducted to differentiate between NTM-sensitivity and NTM-resistance, as well as slowly and rapidly growing mycobacteria. A P-value of less than 0.05 was considered to indicate a significant difference.

## Results

### Baseline information of participants

The baseline information of participants is presented in Table 1. There were 111 cases in the NTM group, with 56 males and 55 females and an average age of 54.67±1.90 years; 50 cases in the TB group, with 31 males and 19 females and an average age of 42.85± 2.48 years; and 40 cases in the HC group, with 24 males and 16 females and an average age of 43.83± 3.06 years. Among the NTM group, 38 cases were urban residents, 81 were smokers, 10 were newly diagnosed, 55 were under treatment, and 46 were not receiving treatment. For the types of NTM, there were 55 cases of *Mycobacterium intracellulare*, 18 cases of *Mycobacterium abscessus*, 12 cases of *Mycobacterium kansasii*, 8 cases of *Mycobacterium chelonae*, 7 cases of *Mycobacterium fortuitum*, 5 cases of *Mycobacterium avium*, and 6 cases of other mycobacteria. There were 38 cases of patients with cavity lesions in the NTM group. Additionally, there were 29 mild, 32 moderate, and 12 severe cases.

**Table 1:** Demographic data of all participants.

Characteristics	NTM (n=111)	Tuberculosis (n=50)	Healthy controls (n=40)
Gender (male/female)	56/55	31/19	24/16
Age, years	54.67±1.90	42.85±2.48	43.83±3.06
Regions			
Urban	38 (34.23%)	16 (14.41%)	25 (22.52%)
Rural	73 (65.76%)	34 (30.63%)	5 (4.50%)
Current smoker	30 (27.02%)	15 (13.51%)	12 (10.81%)
Ex-smoker	81 (72.97%)	35 (31.53%)	28 (34.23%)
Treatment status			
Treatment-naive	10 (9.01%)	20 (18.02%)	N.A.
On therapy	55 (49.55%)	25 (22.52%)	N.A.
Off therapy	46 (41.44%)	5 (4.50%)	N.A.
NTM classification			
*Mycobacterium intracellulare*	55 (49.55%)	N.A.	N.A.
*Mycobacterium abscessus*	18 (16.21%)	N.A.	N.A.
*Mycobacterium kansensis*	12 (10.81%)	N.A.	N.A.
*Mycobacterium chelonis*	8 (7.20%)	N.A.	N.A.
*Mycobacterium fortuitus*	7 (6.30%)	N.A.	N.A.
*Mycobacterium avium*	5 (4.50%)	N.A.	N.A.
Other	6 (5.41%)	N.A.	N.A.
Radiologic features			
Cavity	38 (34.23%)	35 (34.23%)	0
Severity			
Mild	67 (60.36%)	38 (34.23%)	0
Moderate	32 (28.83%)	10 (34.23%)	0
Severe	12 (10.81%)	2 (34.23%)	0
White blood cells (*10^9^/L)	5.80±2.18	6.20±2.35	5.83±1.24
Lymphocytes (*10^9^/L)	1.47±0.71	1.34±0.53	1.88±0.48
Monocytes (*10^9^/L)	0.52±0.25	0.48±0.21	0.32±0.08
Glucose (mg/dL)	5.18±0.18	5.21±0.25	5.19±0.23
Urea (mg/dL)	4.79±0.18	4.97±0.26	4.84±0.19
Creatinine (mg/dL)	59.38±2.09	60.89±2.83	63.58±2.38
AST (U/I)	30.13±6.82	17.10±2.31	16.88±2.29
ALT (U/I)	30.32±4.23	22.50±2.38	21.35±1.28

Data are reported as mean±SD or number and percentage. AST: aspartate transaminase; ALT: alanine transaminase; N.A.: not available; NTM: non-tuberculous mycobacteria; SD: standard deviation.

### Analysis of cytokine levels in the different groups

**Figure 1:**
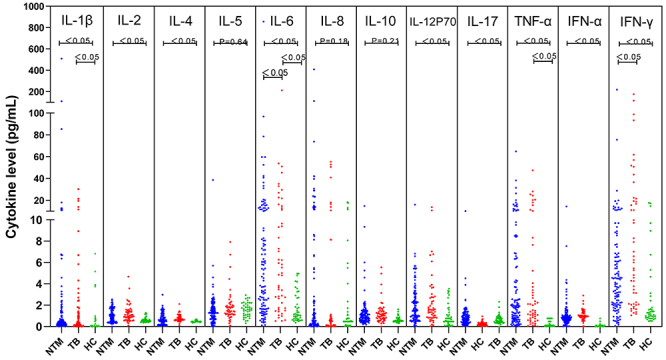
Changes in cytokine levels among the non-tuberculous mycobacteria (NTM), tuberculosis (TB), and healthy control (HC) groups. Data are reported as means±SD. *P<0.05, Kruskal-Wallis test.

We measured the levels of 12 cytokines in the NTM group, TB group, and HC group. As shown in Figure 1, the levels of IL-1β, IL-6, IL-8, TNF-α, and IFN-γ in the serum samples of patients in the NTM and TB groups were elevated, with statistical significance observed only for IL-6 and IFN-γ. The concentration levels of IL-2, IL-4, IL-5, IL-10, IL-12P70, IL-17, and IFN-α were statistically significant in the NTM group and the HC group. Statistical analysis of the subtypes of NTM is presented in Supplementary Table S1.

### Cytokine changes in the NTM group before and after treatment

**Figure 2:**
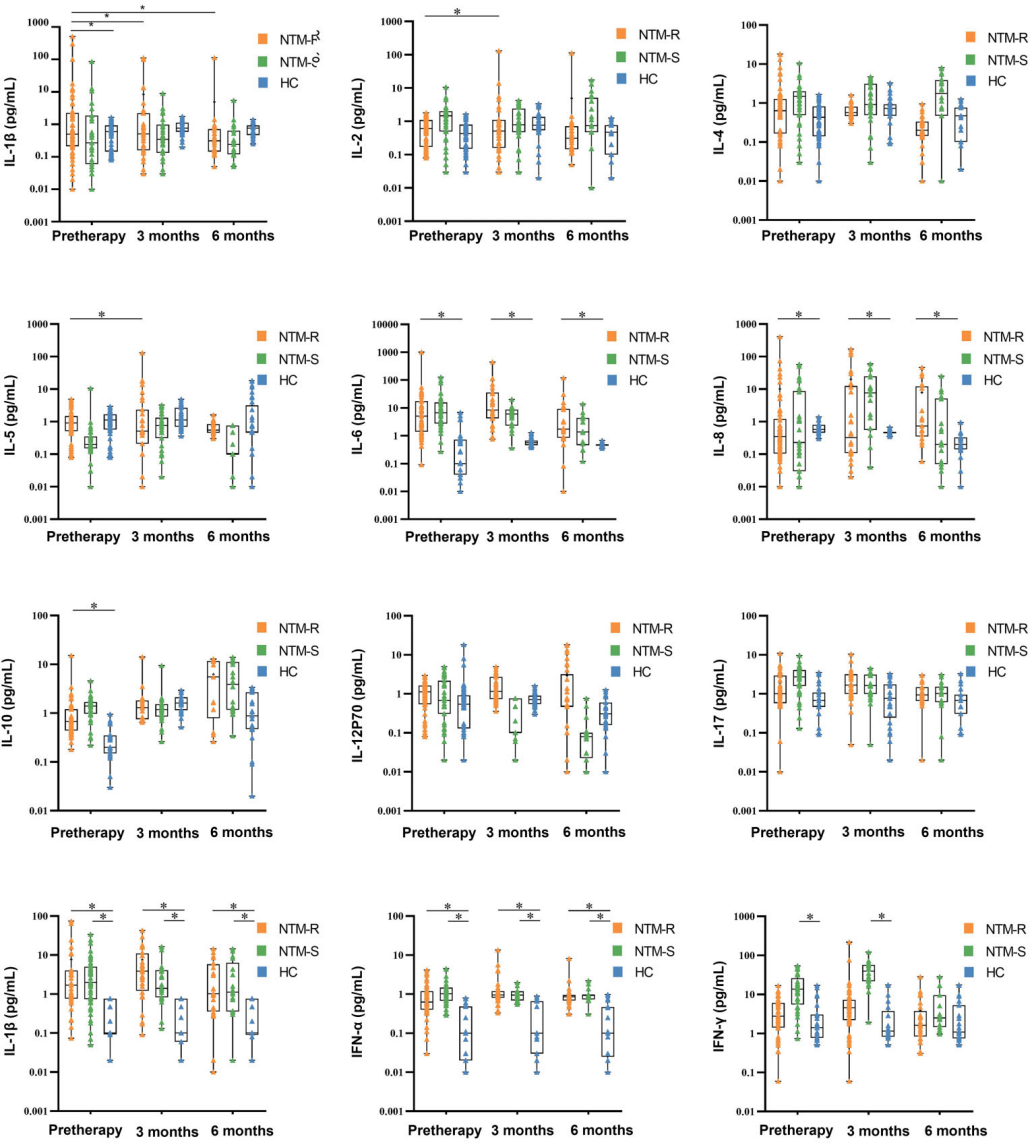
Changes in cytokine levels among the healthy control (HC), the drug-sensitive non-tuberculous mycobacteria (NTM-S), and the drug-resistant NTM (NTM-R) groups before treatment and after 3 and 6 months of treatment. Data are reported as median and IQR. *P<0.05, Kruskal-Wallis test.

The patients in the NTM group were treated according to the national standardized anti-mycobacterial treatment regimen (9). The changes in cytokines before treatment and at 3 and 6 months after treatment were monitored (Figure 2). Among the 111 patients in the NTM group, 68 cases were drug-sensitive (sensitive group) and 43 cases were drug-resistant (resistant group). Compared with before treatment, the level of IL-1β significantly decreased at 3 and 6 months after treatment (P<0.05) (Figure 2). Compared with before treatment and at 6 months after treatment, the IL-6 concentration significantly increased at 3 months after treatment (P<0.05), and the concentration of IL-6 in the resistant group was significantly higher than that in the sensitive group (P<0.05). The IL-10 concentration gradually increased with the prolongation of treatment time. The concentrations of IL-8, TNF-α, and IFN-α in the drug-sensitive and drug-resistant groups were higher than those in the healthy control group (P<0.05). However, there was no significant change in IL-2, IL-4, IL-5, IL-12P70, and IL-17 before and after treatment.

**Figure 3:**
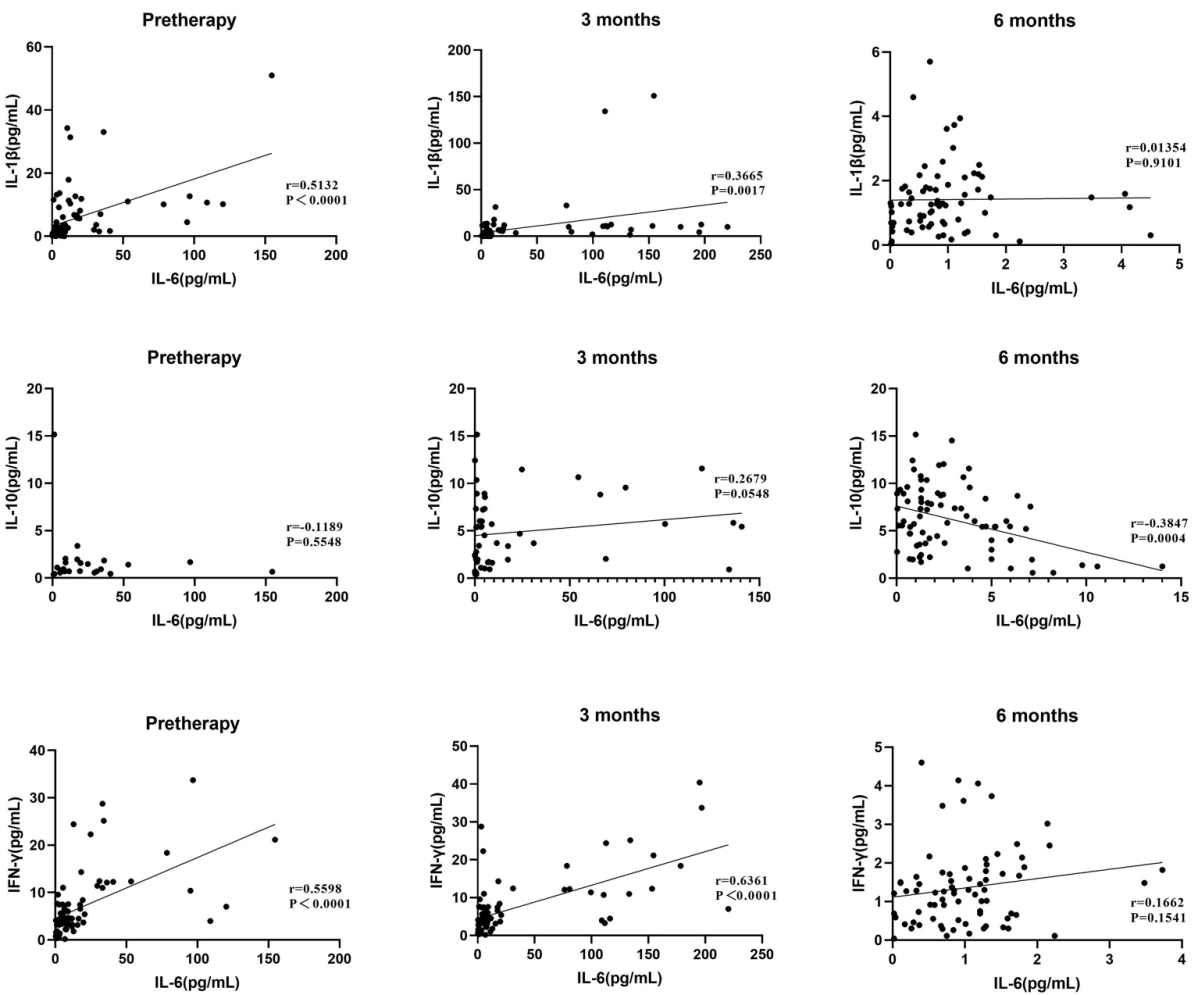
Correlation of interleukin (IL)-6 with IL-1β, IL-10, and interferon (IFN)-γ before treatment and after 3 and 6 months of treatment. P<0.05, Pearson correlation analysis.

To determine treatment efficacy, correlation analysis was performed. Before treatment and at 3 months of treatment, the concentrations of IL-6 and IL-1β were positively correlated (Figure 3). After 6 months of treatment, the concentrations of IL-6 and IL-10 were negatively correlated. Before treatment and at 3 months of treatment, the concentrations of IL-6 and IFN-γ showed a good positive correlation.

### ROC curve analysis of cytokines in differentiating between NTM drug-resistant and drug-sensitive patients, as well as between NTM and TB groups

**Figure 4:**
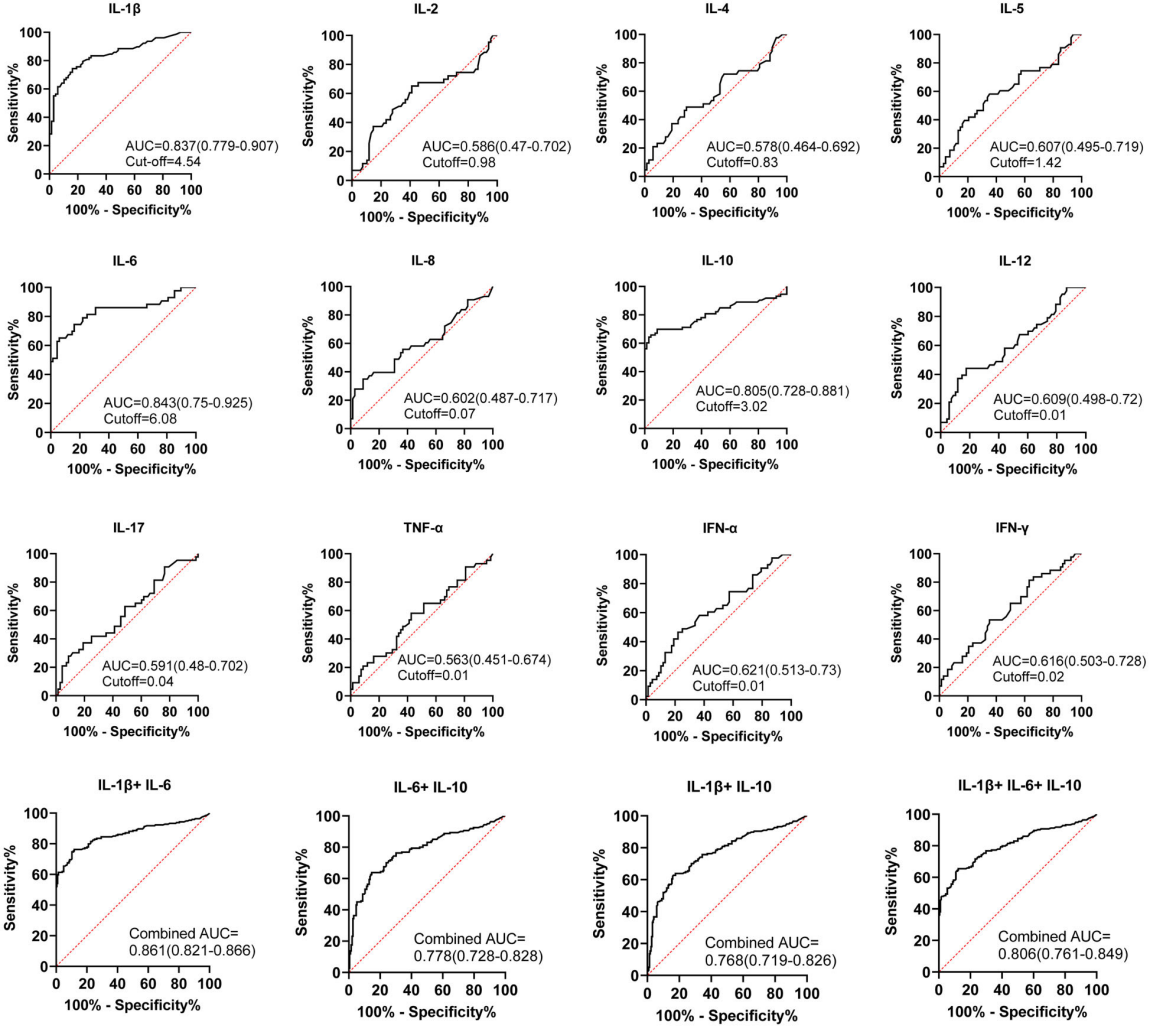
ROC curves of different cytokines in distinguishing drug-resistant group and drug-sensitive group.

To differentiate between NTM drug-resistant and drug-sensitive patients, a ROC curve analysis of cytokines was performed. As shown in Figure 4 and Table 2, IL-6 had an AUC of 0.84 and a cutoff value of 6.08. The AUC for IL-1β was 0.83, and the cutoff value was 4.54. The AUC and cutoff values of IL-10 were 0.81 and 3.02, respectively. These results indicated that IL-6 alone had a high value in distinguishing between NTM drug-resistant and drug-sensitive groups. ROC curve analysis of the combination of IL-1β, IL-6, and IL-10 showed that the AUC for IL-1β+IL-6+IL-10 was 0.81, for IL-1β+IL-6 was 0.86, for IL-6+IL-10 was 0.78, and for IL-1β+IL-10 was 0.77. This indicated that the combination of IL-6 and IL-1β had the highest value in distinguishing NTM-resistant and NTM-sensitive patients.

**Table 2:** ROC data and area under the curve (AUC) of different cytokines in the drug-resistant and drug-sensitive groups.

Cytokines	AUC	Cutoff	Sensitivity (%)	Specificity (%)	Youden index	95%CI	P
IL-1β	0.83	4.54	60.50	69.10	0.35	0.77-0.91	<0.001
IL-2	0.58	0.98	65.10	58.80	0.38	0.47-0.70	<0.001
IL-4	0.57	0.83	48.80	70.60	0.39	0.46-0.69	<0.001
IL-5	0.61	1.42	58.10	64.70	0.37	0.49-0.71	<0.001
IL-6	0.84	6.08	65.10	94.10	0.43	0.75-0.92	<0.001
IL-8	0.59	2.77	34.90	91.20	0.39	0.49-0.72	<0.001
IL-10	0.81	3.02	41.90	88.20	0.40	0.72-0.88	<0.001
IL-12P70	0.60	0.01	44.20	82.40	0.41	0.49-0.72	<0.001
IL-17	0.59	0.04	27.90	91.20	0.44	0.48-0.70	<0.001
TNF-α	0.56	0.01	58.10	57.40	0.35	0.45-0.67	<0.001
IFN-α	0.62	0.01	46.50	76.10	0.38	0.51-0.73	<0.001
IFN-γ	0.61	0.02	80.00	40.30	0.34	0.50-0.72	<0.001
IL-1β+ IL-6	0.86	-	81.90	91.25	0.41	0.82-0.89	<0.001
IL-6+ IL-10	0.77	-	67.19	87.35	0.37	0.72-0.82	<0.001
IL-1β+ IL-10	0.76	-	77.42	78.24	0.38	0.71-0.81	<0.001
IL-1β+ IL-6+ IL-10	0.80	-	79.31	69.34	0.41	0.76-0.84	<0.001

IL: interleukin; TNF: tumor necrosis factor; IFN: interferon.

**Figure 5:**
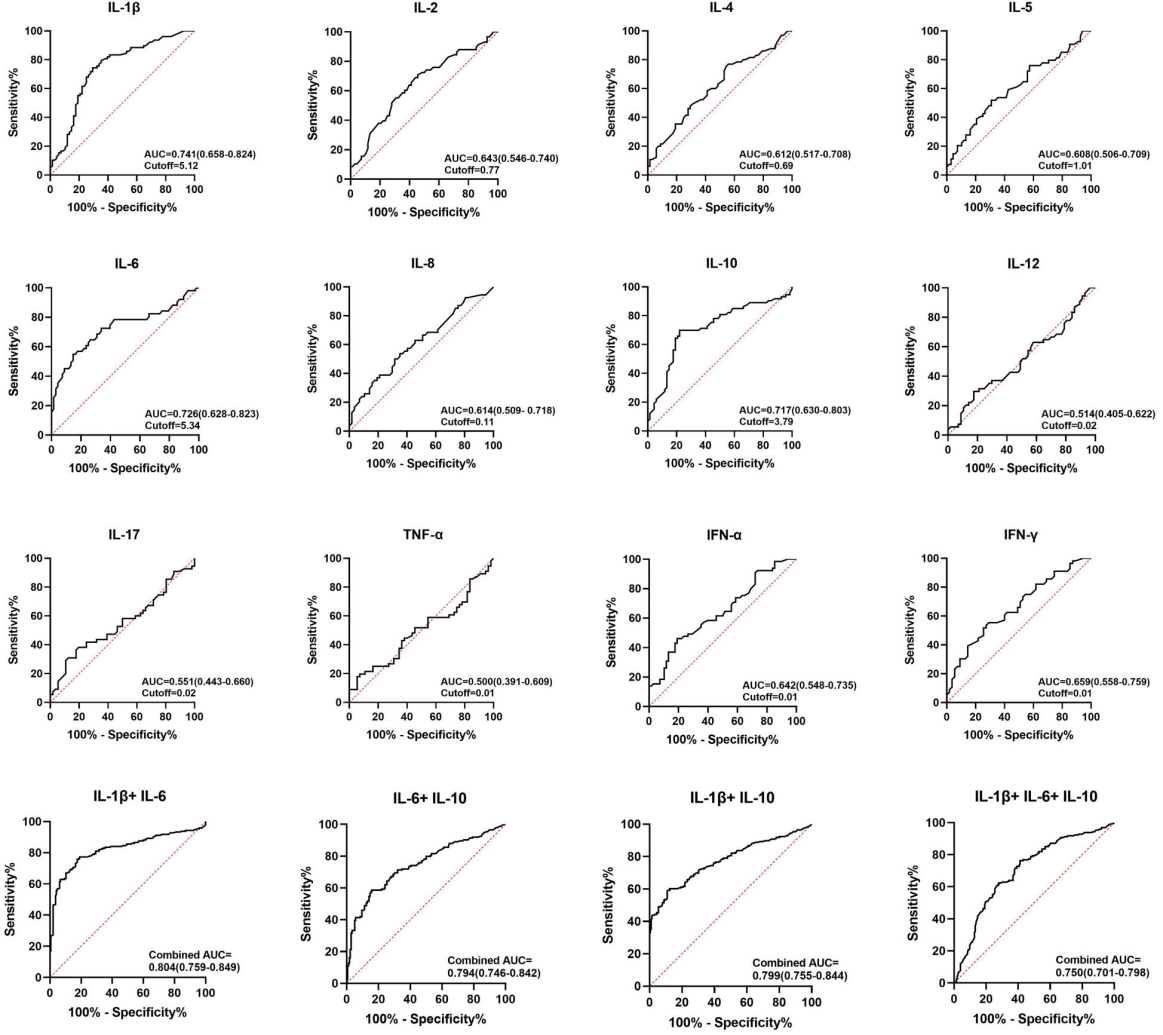
ROC curves of different cytokines in distinguishing non-tuberculous mycobacteria (NTM) and tuberculosis (TB) groups.

Moreover, ROC curve analysis was also conducted on cytokine levels to differentiate between NTM and TB groups. The results, illustrated in Figure 5 and Table 3, revealed that IL-1β had an AUC of 0.74 with a cutoff value of 5.12, while IL-6 showed an AUC of 0.73 with a cutoff value of 5.34. Additionally, IL-10 demonstrated an AUC of 0.72 with a cutoff value of 3.79. These findings suggested that utilizing IL-1β alone is highly effective in distinguishing between NTM and TB groups. Furthermore, the combination of IL-1β, IL-6, and IL-10 was analyzed, with the AUC for IL-1β+IL-6 being 0.80, for IL-1β+IL-10 and IL-6+IL-10 being 0.79, and for IL-1β+IL-6+IL-10 being 0.75. Remarkably, the combination of IL-1β and IL-6 exhibited the greatest discriminatory value between NTM and TB groups.

**Table 3:** ROC data and area under the curve (AUC) of different cytokines in differentiating between non-tuberculous mycobacteria (NTM) and tuberculosis (TB) groups.

Cytokines	AUC	Cutoff	Sensitivity (%)	Specificity (%)	Youden index	95%CI	P
IL-1β	0.74	5.12	68.10	72.53	0.44	0.66-0.82	<0.001
IL-2	0.64	0.77	49.60	58.75	0.35	0.55-0.74	<0.001
IL-4	0.61	0.69	55.14	73.45	0.33	0.52-0.71	<0.001
IL-5	0.61	1.01	47.33	52.17	0.31	0.51-0.71	<0.001
IL-6	0.73	5.34	67.77	87.38	0.49	0.63-0.82	<0.001
IL-8	0.61	0.11	47.89	78.55	0.34	0.51-0.72	<0.001
IL-10	0.72	3.79	67.92	75.14	0.41	0.63-0.80	<0.001
IL-12P70	0.51	0.02	53.77	75.30	0.31	0.49-0.72	<0.001
IL-17	0.55	0.02	37.64	56.27	0.34	0.44-0.66	<0.001
TNF-α	0.50	0.01	61.18	68.70	0.38	0.39-0.61	<0.001
IFN-α	0.64	0.01	56.27	64.26	0.41	0.55-0.74	<0.001
IFN-γ	0.66	0.01	66.17	75.31	0.31	0.56-0.76	<0.001
IL-1β+ IL-6	0.80	-	78.93	88.15	0.48	0.76-0.85	<0.001
IL-6+ IL-10	0.79	-	77.43	79.57	0.46	0.75-0.84	<0.001
IL-1β+ IL-10	0.79	-	79.43	83.24	0.39	0.75-0.84	<0.001
IL-1β+ IL-6+ IL-10	0.75	-	74.47	88.65	0.40	0.70-0.79	<0.001

IL: interleukin; TNF: tumor necrosis factor; IFN: interferon.

### Heat map analysis of cytokines between NTM drug-resistant and drug-sensitive groups

**Figure 6:**
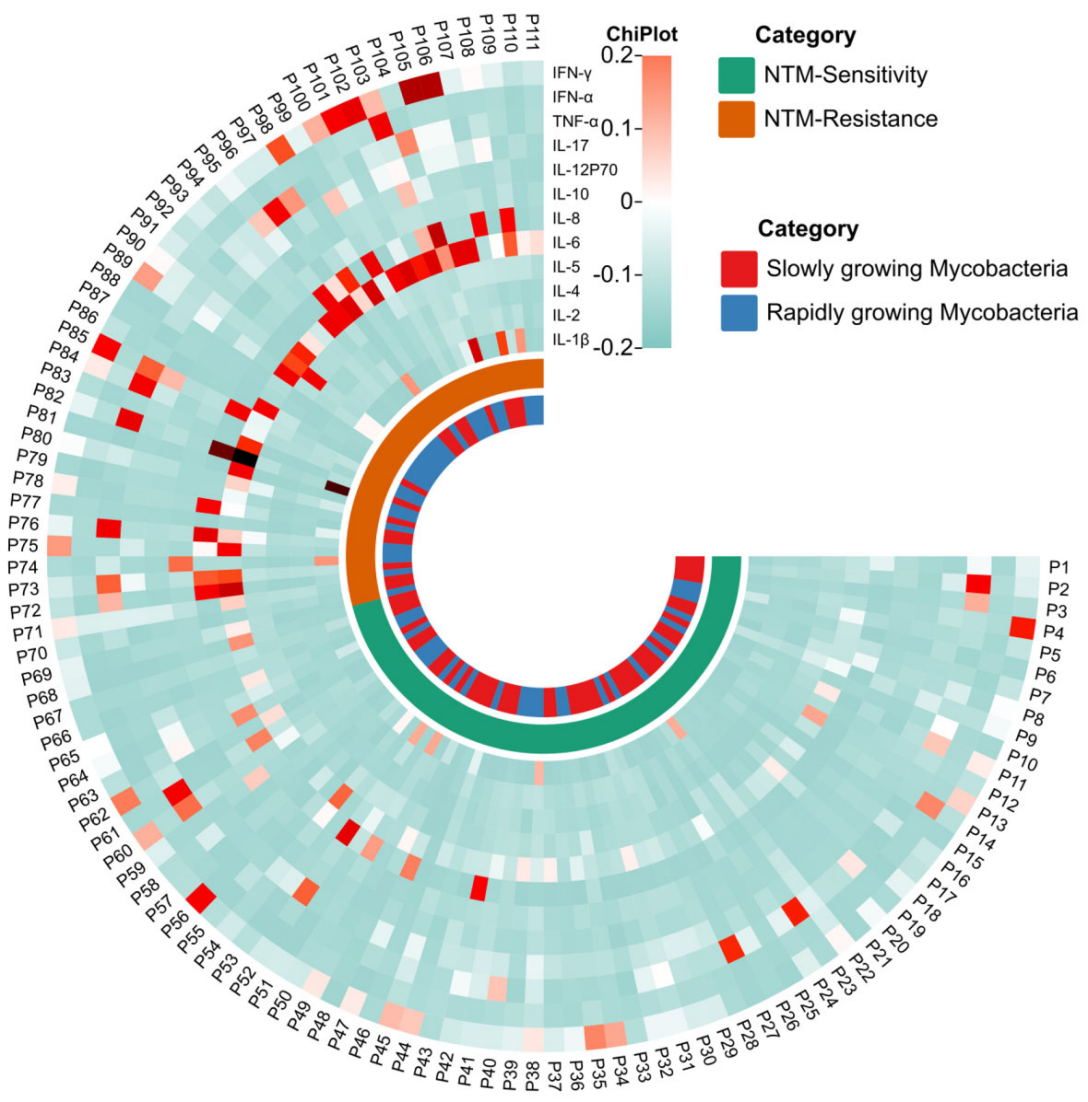
Heat map of cytokine changes in different categories of non-tuberculous mycobacteria (NTM).

The differences in cytokine expression between NTM drug-resistant and drug-sensitive groups were analyzed using heat maps. As shown in Figure 6, the results revealed that the expression levels of IL-6, IL-8, TNF-α, and IFN-γ in NTM patients were higher than those of other cytokines. Among them, the difference in IL-6 was significant between the two groups, with the concentration of IL-6 being higher in the drug-resistant group than in the drug-sensitive group. The fast-growing type of NTM mostly showed drug resistance, whereas the slow-growing type of NTM showed sensitivity. Therefore, the concentration of IL-6 can distinguish between the NTM-resistant group and the NTM-sensitive group.

### Relationship of IL-6/IL-10 ratio with disease severity

**Figure 7:**
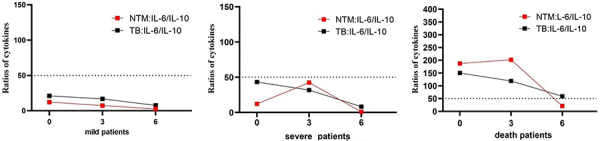
The relationship of interleukin (IL)-6/IL-10 ratio with disease severity.

To determine the relationship of cytokine levels with disease severity, we analyzed the changes in IL-6/IL-10 ratio before treatment and at 3 and 6 months after treatment in both NTM and TB groups. The NTM patients comprised 80 mild cases, 22 severe cases, and 9 deceased cases. The TB patients comprised 28 mild cases, 15 severe cases, and 7 deceased cases. During the 6-month treatment period, mild patients in both NTM and TB groups exhibited consistently low IL-6/IL-10 ratios, with the highest ratio recorded at 12.26 (Figure 7). In contrast, severe cases in NTM and TB groups showed fluctuating IL-6/IL-10 ratios, peaking at 42.41 at 3 months after treatment. Notably, the patients with deceased levels in the NTM and TB groups exhibited markedly elevated IL-6/IL-10 ratios, reaching 202.28 before treatment. Therefore, the IL-6/IL-8 ratio in severe and mild patients of NTM and TB groups was below 50, while in deceased patients, it exceeded 50, suggesting that a high IL-6/IL-8 ratio is associated with increased disease severity.

## Discussion

In recent years, the infection caused by NTM has been gradually increasing, attracting more and more attention (10,11). When NTM invades the body, its pathogenesis is roughly similar to that of *M*. *tuberculosis*, mainly replicating and engulfing within macrophages (12). The normal defense system promotes the production of various cytokines. Some researchers (13) have conducted therapeutic evaluations of cytokines in patients with TB, and the results demonstrate that IL-6 can effectively differentiate between TB patients and healthy individuals. There is also evidence suggesting that IL-6 can serve as a biomarker for treatment and recovery during the course of anti-mycobacterial treatment (14). Therefore, this study aimed to investigate the dynamic changes of cytokines in NTM patients, establish a cytokine model for differentiating NTM patients from healthy individuals, and monitor the therapeutic effects of NTM by assessing changes in cytokine levels. It has been shown (15) that the levels of IL-10 in TB or non-TB patients significantly increases in the early stages of treatment, while in patients with effective treatment, IL-10 decreases. Consistently, our study showed a significant negative correlation between IL-6 and IL-10 after 6 months of treatment. IL-1β and IFN-γ were significantly positively correlated with IL-6 in the early stages of treatment in patients.

IL-1β, IL-6, IL-8, IFN-γ, and TNF-α are all pro-inflammatory factors (16). When the body is infected with mycobacteria or NTM, there is a significant inflammatory response mediated by these cytokines (17). This study found that the levels of IL-1β, IL-6, IL-8, IFN-α, IFN-γ, and TNF-α in the NTM group and the TB group were higher than those in the HC group. Additionally, the concentration of IL-6 in the NTM group was also significantly higher than that in the TB group. IL-6 can promote the production of IFN-γ, enhance the cytotoxic activity of T cells and natural killer cells, and inhibit the growth of *M*. *tuberculosis* (18,19). Some studies have found that the levels of IL-6 and TNF-α inflammatory cells increase in pulmonary tuberculosis (20,21), which is similar to the conclusion of this study. This study analyzed the concentration changes of various cytokines of NTM and *M*. *tuberculosis*, which provides a good complement to the conclusions of previous researchers. Since the single nucleotide polymorphism of the IL-6 cytokine gene is associated with the susceptibility, severity, and clinical outcomes of many infectious diseases (22-24), we also conducted a systematic analysis of the concentration changes of various cytokines in different treatment stages of NTM.

This study analyzed the changes in cytokine levels before treatment and at 3 and 6 months of treatment in NTM patients. The results revealed that in the drug-sensitive group, the levels of pro-inflammatory cytokines IL-1β, IL-6, and IL-17 decreased with the progression of the treatment period, while the anti-inflammatory cytokine IL-10 increased, indicating that both pro-inflammatory and anti-inflammatory cytokines play a role in patients undergoing anti-mycobacterial drug treatment. The increase in IL-10 may also be due to reduced original lymphocyte response and decreased IFN-γ in patients with active NTM (25). In the drug-resistant group, it was found that the concentration of IL-6 was highest at 3 months of treatment, indicating an imbalance between pro-inflammatory and anti-inflammatory responses after anti-mycobacterial drug treatment. The IL-6 inflammatory regulatory mechanism is more likely to be activated in drug-resistant patients, whereas IFN-γ induces this phenomenon in the drug-sensitive group, aligning with Ocaãa-Guzman et al. research findings (26). Ponnana et al. (27) indicated that IL-6 promoted IFN-γ secretion, thereby enhancing the killing ability of natural killer cells and further inhibiting the growth of NTM. Additionally, this study conducted a heat map analysis of the average cytokine levels in 111 NTM patients, which visually showed that IL-6 was positively correlated in the drug-resistant group, further confirming the reliability of the results of this study.

Significant differences were observed in the levels of most cytokines in the plasma of patients in different groups, including the NTM drug-resistant and drug-sensitive groups, as well as the NTM and TB groups. To explore their discriminatory potential, a ROC analysis was conducted. The results indicated that the combination of IL-6 and IL-1β accurately distinguished between NTM drug-resistant and drug-sensitive groups, with an AUC of 0.86, while for NTM and TB groups, the combination of IL-6 and IL-1β demonstrated an AUC of 0.80. Therefore, combining these cytokines shows promise in improving the differentiation of NTM drug-resistant groups and distinguishing between NTM and TB groups.

Furthermore, our results showed that patients with an IL-6/IL-10 ratio of less than 50 had a good prognosis, suggesting that the IL-6/IL-10 ratio before treatment may be used as a predictive marker of disease prognosis. However, these results were preliminary, and further studies are needed to validate our findings.

The current study had certain limitations that are worth noting. First, the sample size was limited. Second, NTM consists of numerous species. The study did not conduct subgroup analysis based on various species. We will address these limitations in future research.

In summary, this study demonstrated that IL-6 and IL-10 may serve as biomarkers for early treatment efficacy and prognosis of NTM. Cytokine analysis helped distinguishing between drug-resistant and drug-sensitive NTM patients as well as differentiating NTM from TB groups. Particularly, the combination of IL-6 and IL-1β had the highest discriminatory value. Additionally, the IL-6/IL-10 ratio was associated with disease severity, showing higher ratios in deceased patients. The findings indicated that targeting IL-6 and IL-10 could actively manage NTM infection and facilitate early intervention to mitigate excessive cytokine-induced inflammation in the human body.

## Supplementary Material

Click here to view [pdf].

## References

[1] Pai M, Behr MA, Dowdy D, Dheda K, Divangahi M, Boehme CC (2016). Tuberculosis. Nat Rev Dis Primers.

[2] Daley CL, Iaccarino JM, Lange C, Cambau E, Wallace RJ, Andrejak C (2020). Treatment of nontuberculous mycobacterial pulmonary disease: an official ATS/ERS/ESCMID/IDSA clinical practice guideline. Eur Respir J.

[3] Haworth CS, Banks J, Capstick T, Fisher AJ, Gorsuch T, Laurenson IF (2017). British Thoracic Society guidelines for the management of non-tuberculous mycobacterial pulmonary disease (NTM-PD). Thoraxb.

[4] Cadena AM, Fortune SM, Flynn JL (2017). Heterogeneity in tuberculosis. Nat Rev Immunol.

[B05] O'Garra A, Redford PS, McNab FW, Bloom CI, Wilkinson RJ, Berry MPR (2013). The immune response in tuberculosis. Annu Rev Immunol.

[6] Kumar NP, Moideen K, Banurekha VV, Nair D, Babu S (2019). Plasma proinflammatory cytokines are markers of disease severity and bacterial burden in pulmonary tuberculosis. Open Forum Infect Dis.

[7] Yu F, Chen X, Ji Z, Xu K (2016). Prevalence of nontuberculous mycobacteria in Hangzhou during 2009-2014 [in Chinese]. Chin J Microecol.

[8] The Chinese Medical Association Tuberculosis Branch (2020). Guidelines for the Diagnosis and Treatment of NTM Disease (2020). Chin J Tuberculosis Respir.

[9] Zhang P (2001). Guidelines for the diagnosis and treatment of pulmonary tuberculosis [in Chinese]. Chin J Tuberculosis Respir Dis.

[10] Wu TS, Lu CC, Lai HC (2009). Current situations on identification of nontuberculous mycobacteria. J Biomed Lab Sci.

[11] Moore JE, Kruijshaar ME, Ormerod LP, Drobniewski F, Abubakar I (2010). Increasing reports of non-tuberculous mycobacteria in England, Wales and Northern Ireland, 1995-2006. BMC Public Health.

[12] Henkle E, Winthrop KL (2015). Nontuberculous mycobacteria infections in immunosuppressed hosts. Clin Chest Med.

[13] Mencarini J, Cresci C, Simonetti MT, Truppa C, Camiciottoli G, Frilli ML (2017). Non-tuberculous mycobacteria: epidemiological pattern in a reference laboratory and risk factors associated with pulmonary disease. Epidemiol Infect.

[14] Furuuchi K, Morimoto K, Yoshiyama T, Tanaka Y, Fujiwara K, Okumura M (2019). Interrelational changes in the epidemiology and clinical features of nontuberculous mycobacterial pulmonary disease and tuberculosis in a referral hospital in Japan. Respir Med.

[15] Zhu XY, Chen H, Xu JC, Wu MY, Chen XN, Xu P (2015). Significance of sIL-2R, IL-6, IL-8, IL-10 and TNF-α expression in peripheral blood of patients with tuberculosis [in Chinese]. J Clin Pulm Med.

[16] Winthrop KL, Marras TK, Adjemian J, Zhang H, Wang P, Zhang Q (2020). Incidence and prevalence of nontuberculous mycobacterial lung disease in a large U.S. managed care health plan, 2008-2015. Ann Am Thorac Soc.

[17] Zhang J, Su JR, Ding BC, Liu JW, Yi JL, Yang XY (2017). Distribution and drug resistance of nontuberculous mycobacteria in Beijing [in Chinese]. Zhonghua Jie He He Hu Xi Za Zhi.

[18] Dorhoi A, Kaufmann SH (2016). Pathology and immune reactivity: understanding multidimensionality in pulmonary tuberculosis. Semin Immunopathol.

[19] Shao Y, Chen C, Song H, Li G, Liu Q, Li Y (2015). The epidemiology and geographic distribution of nontuberculous mycobacteria clinical isolates from sputum samples in the eastern region of China. PLoS Negl Trop Dis.

[20] Aravindhan V, Kevinkumar V, Dhamodharan U, Viswanathan V (2018). Serum levels of chemokines IP-10, IL-8 and SDF-1 serve as good biomarkers for diabetes-tuberculosis nexus. J Diabetes Complications.

[21] Wu J, Zhang Y, Li J, Lin S, Wang L, Jiang Y (2014). Increase in nontuberculous mycobacteria isolated in Shanghai, China: results from a population-based study. PLoS One.

[22] Roberts T, Beyers N, Aguirre A, Walzl G (2007). Immunosuppression during active tuberculosis is characterized by decreased interferon-gamma production and CD25 expression with elevated forkhead box P3, transforming growth factor-beta, and interleukin-4 mRNA levels. J Infect Dis.

[B23] Yu X, Liu P, Liu G, Zhao L, Hu Y, Wei G (2016). The prevalence of non-tuberculous mycobacterial infections in mainland China: systematic review and meta-analysis. J Infect.

[24] Zhang ZX, Cherng BPZ, Sng LH, Tan YE (2019). Clinical and microbiological characteristics of non-tuberculous mycobacteria diseases in Singapore with a focus on pulmonary disease, 2012-2016. BMC Infect Dis.

[25] Richard M, Gutiérrez AV, Kremer L (2020). Dissecting erm(41)-mediated macrolide-inducible resistance in *Mycobacterium abscessus*. Antimicrob Agents Chemother.

[26] Ocaãa-Guzman R, Tellez-Navarrete NA, Preciado-Garcia M, Ponce-Gallegos MA, Buendia-Roldan I, Falfán-Valencia R (2021). Multidrug-resistant tuberculosis patients expressing the HLA-DRB1*04 allele, and after treatment they show a low frequency of HLA-II+ monocytes and a chronic systemic inflammation. Microb Pathog.

[27] Ponnana M, Sivangala R, Joshi L, Valluri V, Gaddam S (2017). IL-6 and IL-18 cytokine gene variants of pulmonary tuberculosis patients with co-morbid diabetes mellitus and their household contacts in Hyderabad. Gene.

