# Supplementary material for: Plasma cytokine levels as markers of pathogenesis and treatment response in patients with non-tuberculous mycobacterial pulmonary disease
Source: Braz J Med Biol Res. 2024 Sep 6;57:e13755. doi: 10.1590/1414-431X2024e13755 (PMC11379429; doi:10.1590/1414-431X2024e13755)
Supplement: Supplementary file 1 [file 1414-431X-bjmbr-57-e13755-suppl.pdf]

Table S1. Cytokine levels of different types of non-tuberculous mycobacteria (NTM) and Runyon classification of NTM.

Cytokines (pg/mL)	NTM classification							Runyon classification of NTM			
	M. intracellulare	*M. abscessus*	*M. kansensis*	*M. chelonis*	*M. fortuitus*	*M. avium*	Other	Slowly growing Mycobacteria			Rapidly growing mycobacteria
								Slowly growing photochromogenic mycobacteria	Slowly growing dark colored mycobacteria	Slowly growing non-chromogenic mycobacteria	
IL-1β	4.14 (0.01–19.35)	6.05 (0.21–30.42)	7.79 (0.10–9.84)	5.49 (1.10–8.89)	11.49 (2.10–509.39)	8.40 (0.19–15.489)	6.71 (1.70–11.39)	5.21 (1.40–10.39)	7.53 (2.77–8.41)	4.56 (0.79–7.38)	9.77 (3.19–15.37)
IL-2	1.54 (0.14–2.09)	1.93 (0.45–2.39)	1.48 (0.23–2.74)	1.03 (0.47–2.30)	1.13 (0.27–7.13)	0.78 (0.20–1.73)	1.48 (0.23–2.74)	1.16 (0.27–2.48)	1.36 (0.13–1.77)	1.79 (0.93–2.88)	0.83 (0.31–1.31)
IL-4	1.09 (0.42–1.79)	1.02 (0.22–2.10)	1.39 (0.08–0.69)	1.02 0.22–2.10)	1.42 (0.62–2.19)	1.72 (1.22–3.15)	1.00 (0.62–2.79)	1.02 (0.43–3.03)	0.93 (0.22–2.35)	1.58 (0.48–2.37)	1.22 (0.42–3.15)
IL-5	1.32 (0.52–3.98)	3.94 (0.21–38.98)	1.55 (0.08–12.51)	3.05 (1.08–10.50)	2.05 (0.22–7.59)	1.05 (0.08–9.10)	4.75 (2.08–14.19)	3.55 (0.49–5.61)	2.15 (0.08–7.38)	3.23 (1.22–6.67)	4.22 (1.21–39.98)
IL-6	38.7 (3.11–885.87)	24.26 (4.50–84.26)	34.21 (2.59–74.46)	22.22 (2.36–44.76)	30.26 (0.50–54.26)	28.66 (2.89–74.16)	39.36 (1.50–93.26)	17.34 (4.72–54.88)	33.17 (0.50–44.76)	36.78 (2.89–885.87)	38.73 (5.16–74.16)
IL-8	11.5 (0.01–408.40)	15.67 (4.01–35.14)	12.56 (3.51–18.27)	7.05 (1.21–80.42)	12.61 (2.11–30.74)	5.87 (2.09–15.14)	11.67 (3.41–45.64)	14.70 (0.01–35.14)	9.92 (2.36–408.40)	15.41 (3.49–80.42)	12.19 (2.36–41.15)
IL-10	3.16 (2.24–6.67)	2.31 (0.29–14.67)	1.66 (0.33–2.61)	1.45 (0.11–6.69)	0.81 (0.42–1.70)	1.01 (0.29–1.10)	1.12 (0.32–3.17)	3.39 (1.29–14.67)	1.39 (0.11–6.69)	1.01 (0.21–4.60)	0.87 (0.89–11.37)
IL-12P70	1.49 (0.27–17.88)	0.99 (0.19–16.21)	1.01 (0.07–5.21)	2.01 (1.04–7.29)	1.71 (0.23–11.25)	0.91 (0.31–2.26)	1.01 (0.09–5.21)	1.17 (0.67–5.57)	1.77 (1.04–7.29)	3.01 (0.07–17.88)	2.25 (0.67–5.21)
IL-17	1.26 (0.41–14.07)	2.26 (0.91–15.67)	3.26 (1.43–6.07)	1.36 (0.21–4.09)	2.16 (0.49–8.02)	1.47 (0.36–10.01)	0.78 (0.01–4.07)	1.38 (0.21–15.67)	1.86 (0.49–10.01)	1.16 (0.01–8.02)	1.67 (0.91–8.02)
TNF-α	3.79 (1.47–64.84)	5.76 (1.51–49.47)	6.69 (0.91–35.79)	3.11 (1.59–54.42)	5.89 (0.51–24.37)	4.15 (0.51–9.47)	5.04 (1.59–19.40)	3.96 (1.47–49.47)	6.71 (1.59–54.52)	4.01 (0.51–9.47)	5.09 (1.50–19.47)
IFN-α	2.07 (0.03–19.10)	1.24 (0.02–2.90)	0.77 (0.01–1.76)	2.17 (0.61–5.89)	1.71 (1.01–3.26)	2.13 (0.91–2.19)	1.24 (0.51–18.76)	2.73 (0.03–3.26)	1.43 (0.61–2.19)	0.89 (0.51–18.76)	2.88 (0.02–19.10)
IFN-γ	18.13 (5.06–218.40)	4.12 (1.04–36.79)	7.18 (4.47–57.58)	9.28 (1.41–17.58)	7.28 (3.11–17.58)	9.19 (5.01–37.50)	15.18 (1.37–37.58)	11.25 (1.04–57.58)	9.21 (3.11–218.40)	14.58 (5.01–37.50)	12.37 (2.89–36.79)

Data are reported as median (Q25–Q75). Slowly growing photochromogenic Mycobacteria included *M. kansasii*, *M. marinum*, and *M. simiae complex*. Slowly growing dark colored Mycobacteria included *M. scrofulaceum* and *M. gordonae*. Slowly growing non-chromogenic Mycobacteria included *M. avium complex* and *M. haemophilum*; Rapidly growing Mycobacteria included *M. smegmatis*, *M. fortuitum*, *M. chelonae*, and *M. mucogenicum*.
